# Supplementary material for: Move-It: A Cluster-Randomised Digital Worksite Exercise Intervention in China: Outcome and Process Evaluation
Source: Int J Environ Res Public Health. 2019 Sep 17;16(18):3451. doi: 10.3390/ijerph16183451 (PMC6766073; doi:10.3390/ijerph16183451)
Supplement: Supplementary file 1 [file ijerph-16-03451-s001.zip › Supplementary files/Supplementary File 3_Statistical tests and power analysis.pdf]

1. Skewness tests
2. Tests of attrition for the 3 baseline outcomes
3. Illustrative example of how FIML works
4. Latent change score Stata model results
5. Mixture/latent class SKEWT illustration from Mplus
6. Power analysis exhibit from https://clincalc.com/stats/samplesize.aspx
7. Test of differences in attrition from https://www.medcalc.org/calc/comparison_of_proportions.php

1. Skewness tests

```
                    Skewness/Kurtosis tests for Normality
                                                          ------ joint ------
    Variable |        Obs  Pr(Skewness)  Pr(Kurtosis) adj chi2(2)   Prob>chi2
-------------+---------------------------------------------------------------
    totmeth1 |        282     0.0000        0.0000           .         0.0000
    totmeth2 |        214     0.0000        0.0033        40.26        0.0000
    totsith1 |        277     0.0016        0.0000        26.40        0.0000
    totsith2 |        204     0.0087        0.0000        21.22        0.0000
    jobperf1 |        277     0.0001        0.0690        15.68        0.0004
    jobperf2 |        211     0.0000        0.0241        24.66        0.0000
```

2. Tests of attrition for the 3 baseline outcomes

2.i. ttest totmeth1 , by(dropped)

```
Two-sample t test with equal variances
------------------------------------------------------------------------------
   Group |     Obs        Mean    Std. Err.   Std. Dev.   [95% Conf. Interval]
---------+--------------------------------------------------------------------
       0 |     214     22.0761    1.547672    22.64049    19.02538    25.12681
       1 |      68    20.15502     2.02663    16.71202    16.10985     24.2002
---------+--------------------------------------------------------------------
combined |     282    21.61286    1.271369    21.34991    19.11024    24.11548
---------+--------------------------------------------------------------------
    diff |            1.921074     2.97516               -3.935447    7.777594
------------------------------------------------------------------------------
    diff = mean(0) - mean(1)                                      t =   0.6457
Ho: diff = 0                                     degrees of freedom =      280

 Pr(T < t) = 0.7405         Pr(|T| > |t|) = 0.5190          Pr(T > t) = 0.2595
   Ha: diff < 0                 Ha: diff != 0                 Ha: diff > 0
```

2.ii. ttest totsith1 , by(dropped)

```
Two-sample t test with equal variances
------------------------------------------------------------------------------
   Group |     Obs        Mean    Std. Err.   Std. Dev.   [95% Conf. Interval]
---------+--------------------------------------------------------------------
       0 |     210    9.442857    .1653386    2.395984    9.116912    9.768802
       1 |      67    9.328358    .3704289    3.032091    8.588773    10.06794
---------+--------------------------------------------------------------------
combined |     277    9.415162    .1537501    2.558912    9.112491    9.717834
---------+--------------------------------------------------------------------
    diff |            .1144989    .3596304               -.5934794    .8224773
------------------------------------------------------------------------------
    diff = mean(0) - mean(1)                                      t =   0.3184
Ho: diff = 0                                     degrees of freedom =      275

    Ha: diff < 0                 Ha: diff != 0                 Ha: diff > 0
 Pr(T < t) = 0.6248         Pr(|T| > |t|) = 0.7504          Pr(T > t) = 0.3752
```

2.iii. ttest jobperf1 , by(dropped)

```
Two-sample t test with equal variances
------------------------------------------------------------------------------
   Group |     Obs        Mean    Std. Err.   Std. Dev.   [95% Conf. Interval]
```

```
---------+--------------------------------------------------------------------
       0 |     211    7.251185    .0923241    1.341086    7.069184    7.433186
       1 |      66    6.681818    .2084006    1.693055    6.265613    7.098023
---------+--------------------------------------------------------------------
    diff |            .5693667     .201977                .1717491    .9669842
------------------------------------------------------------------------------
    diff = mean(0) - mean(1)                                      t =   2.8190
Ho: diff = 0                                     degrees of freedom =      275

    Ha: diff < 0                 Ha: diff != 0                 Ha: diff > 0
 Pr(T < t) = 0.9974         Pr(|T| > |t|) = 0.0052          Pr(T > t) = 0.0026

    Variable |        Obs        Mean    Std. Dev.       Min        Max
-------------+---------------------------------------------------------
  PostMPAMET |        143    297.4825    453.3436          0       2520
```

3. Illustrative example of how FIML works

3.i. A simple summary: parameter estimates for 1 outcome pre and post:

ec: SAMPLE SIZE FOR POST IS 142 ONLY

```
.  sum totmeth1 totmeth2 if group ==1
    Variable |        Obs        Mean    Std. Dev.       Min        Max
-------------+---------------------------------------------------------
    totmeth1 |        196     22.8708    21.90028          0        119
    totmeth2 |        142    27.75332    26.07248          0        119
```

EC: SAMPLE SIZE FOR PAIRED T-TEST DROPS TO POST SAMPLE SIZE 142

```
.  ttest totmeth1=totmeth2 if group ==1
```

ec: SAMPLE SIZE FOR BOTH IS 142 ONLY, SO THE BASELINE MEAN NOW SHIFTED 24.12038 >22.8708

```
Paired t test
------------------------------------------------------------------------------
Variable |     Obs        Mean    Std. Err.   Std. Dev.   [95% Conf. Interval]
---------+--------------------------------------------------------------------
totmeth1 |     142    24.12038    1.975248     23.5378    20.21545    28.02531
totmeth2 |     142    27.75332    2.187954    26.07248    23.42789    32.07876
---------+--------------------------------------------------------------------
    diff |     142   -3.632946    2.231634    26.59299   -8.044734    .7788419
------------------------------------------------------------------------------
     mean(diff) = mean(totmeth1 - totmeth2)                       t =  -1.6279
 Ho: mean(diff) = 0                              degrees of freedom =      141

 Ha: mean(diff) < 0           Ha: mean(diff) != 0           Ha: mean(diff) > 0
 Pr(T < t) = 0.0529         Pr(|T| > |t|) = 0.1058          Pr(T > t) = 0.9471
```

3.ii. A SEM non-FIML similar descriptive model

```
. sem totmeth1 totmeth2 if group ==1
(54 observations with missing values excluded)
```

EC: SEM DROPS THE MISSING POST CASES AND USES ONLY 142 FOR THE ENTIRE MODEL: HENCE THE MEAN ESTIMATE FOR BASELINE FOR WHICH ALL CASES HAD VALUES NOW WILL BE ONLY BASED ON THE 142, NOT 196: SO THE MEAN ESTIMATES DIFFER, SEE BLUE HIGHLIGHTS: 24.12038 > 22.8708

```
Exogenous variables
Observed:  totmeth1 totmeth2
Fitting target model:
Iteration 0:   log likelihood = -1299.0972
Iteration 1:   log likelihood = -1299.0972
Structural equation model                       Number of obs     =        142
Estimation method  = ml
Log likelihood     = -1299.0972
------------------------------------------------------------------------------
                      |                 OIM
                      |      Coef.   Std. Err.      z    P>|z|     [95% Conf. Interval]
----------------------+-------------------------------------------------------
        mean(totmeth1)|   24.12038   1.968281    12.25   0.000     20.26262    27.97813
        mean(totmeth2)|   27.75332   2.180236    12.73   0.000     23.48014    32.02651
```

```
---------------------+----------------------------------------------------------------
         var(totmeth1)|   550.1263   65.28798                          435.9567    694.1949
         var(totmeth2)|    674.987   80.10622                          534.9047    851.7546
---------------------+----------------------------------------------------------------
cov(totmeth1,totmeth2)|   261.4532   55.64512     4.70   0.000     152.3908    370.5156
--------------------------------------------------------------------------------------
LR test of model vs. saturated: chi2(0)   =      0.00, Prob > chi2 =      .
```

3.iii. A FIML SEM model

```
. sem totmeth1 totmeth2 if group ==1,  method(mlmv)
```

EC: SEM WITH FIML USES NOW ALL 196 CASES TO ESTIMATE BOTH PRE AND POST MEANS AND VARIANCES, SO N=196: SO THE BASELINE MEAN ESTIMATES ARE THE SAME, SEE BLUE HIGHLIGHTS ABOVE (THE I. SUMMARY ESTIMATES): 22.8708 = 22.8708

```
Exogenous variables
Observed:  totmeth1 totmeth2
Fitting saturated model:
Iteration 0:   log likelihood = -1533.3765
Iteration 1:   log likelihood = -1532.3527
Iteration 2:   log likelihood = -1532.1521
Iteration 3:   log likelihood =  -1532.152
Fitting baseline model:
Iteration 0:   log likelihood = -1546.6569
Iteration 1:   log likelihood = -1546.5972
Iteration 2:   log likelihood = -1546.5971
Fitting target model:
Iteration 0:   log likelihood =  -1532.152
Iteration 1:   log likelihood =  -1532.152

Structural equation model                      Number of obs     =        196
Estimation method  = mlmv
Log likelihood     =  -1532.152
--------------------------------------------------------------------------------------
                      |                 OIM
                      |      Coef.   Std. Err.      z    P>|z|     [95% Conf. Interval]
---------------------+----------------------------------------------------------------
        mean(totmeth1)|    22.8708    1.56031    14.66   0.000     19.81265    25.92895
        mean(totmeth2)|   27.15945    2.10696    12.89   0.000     23.02988    31.28901
---------------------+----------------------------------------------------------------
         var(totmeth1)|   477.1751   48.20197                          391.4655    581.6506
         var(totmeth2)|   658.5094   76.42463                          524.5345    826.7037
---------------------+----------------------------------------------------------------
cov(totmeth1,totmeth2)|   226.7824   46.15245     4.91   0.000     136.3253    317.2395
--------------------------------------------------------------------------------------
LR test of model vs. saturated: chi2(0)   =      0.00, Prob > chi2 =      .
```

3.iv. A SEM (with Stata gsem) SEM model – default is FIML for gsem!!!

```
. gsem (-> totmeth1 totmeth2) if group ==1
```

EC: gsem LISTS THE SAMPLES WITH VALID SCORES IN EACH VARIABLE BUT USES AS DEFAULT FIML AND HENCE THE ENTIRE SAMPLE TO ESTIMATE ALL PARAMETERS, LIKE SEM WITH method(mlmv)

```
Iteration 0:   log likelihood = -1546.5971
Iteration 1:   log likelihood = -1546.5971

Generalized structural equation model          Number of obs     =        196

Response       : totmeth1                      Number of obs     =        196
Family         : Gaussian
Link           : identity

Response       : totmeth2                      Number of obs     =        142
Family         : Gaussian
Link           : identity
Log likelihood = -1546.5971
--------------------------------------------------------------------------------
```

```
                |      Coef.   Std. Err.      z    P>|z|     [95% Conf. Interval]
----------------+----------------------------------------------------------------
totmeth1        |
          _cons |    22.8708    1.56031    14.66   0.000     19.81265    25.92895
----------------+----------------------------------------------------------------
totmeth2        |
          _cons |   27.75332   2.180236    12.73   0.000     23.48014    32.02651
----------------+----------------------------------------------------------------
 var(e.totmeth1)|   477.1751   48.20197                      391.4655    581.6506
 var(e.totmeth2)|    674.987   80.10622                      534.9047    851.7546
---------------------------------------------------------------------------------
```

4. Latent change score Stata model results:

Physical activity

```
. sem (latentch21 ctotmeth1@1 _cons@0 ->ctotmeth2) (ctotmeth1 _cons ->latentch21),
group(group) ginvariant(n
> one) nocapslatent latent(latentch21)  var(e.ctotmeth2@0 e.latentch21 ) method(mlmv)
showginvariant

Endogenous variables

Observed:  ctotmeth2
Latent:    latentch21

Exogenous variables

Observed:  ctotmeth1
Structural equation model                        Number of obs     =        282
Grouping variable  = group                       Number of groups  =          2
Estimation method  = mlmv
Log likelihood     = -2259.5882

 ( 1)  [ctotmeth2]1bn.group#c.latentch21 = 1
 ( 2)  [ctotmeth2]1bn.group#c.ctotmeth1 = 1
 ( 3)  [/]var(e.ctotmeth2)#1bn.group = 0
 ( 4)  [ctotmeth2]1bn.group = 0
 ( 5)  [ctotmeth2]2.group#c.latentch21 = 1
 ( 6)  [ctotmeth2]2.group#c.ctotmeth1 = 1
 ( 7)  [/]var(e.ctotmeth2)#2.group = 0
 ( 8)  [ctotmeth2]2.group = 0
---------------------------------------------------------------------------------
                 |                 OIM
                 |      Coef.   Std. Err.      z    P>|z|     [95% Conf. Interval]
-----------------+---------------------------------------------------------------
Structural       |
  ctotmeth2      |
      latentch21 |
             [*] |          1  (constrained)
       ctotmeth1 |
             [*] |          1  (constrained)
           _cons |
             [*] |          0  (constrained)
  ---------------+---------------------------------------------------------------
  latentch21     |
       ctotmeth1 |
      Guangzhou  |  -.5247397   .0839641    -6.25   0.000    -.6893062   -.3601731
        Beijing  |  -.8910333   .1797252    -4.96   0.000    -1.243288   -.5387785
           _cons |
      Guangzhou  |    5.79507   1.999515     2.90   0.004     1.876092    9.714048
        Beijing  |   7.409048    3.64259     2.03   0.042     .2697015    14.54839
-----------------+---------------------------------------------------------------
 var(e.ctotmeth2)|
             [*] |          0  (constrained)
var(e.latentch21)|
```

```
      Guangzhou  |   550.7286   65.35945                        436.434     694.955
        Beijing  |   946.4345   157.7385                        682.691     1312.07
---------------------------------------------------------------------------------
Note: [*] identifies parameter estimates constrained to be equal across groups.
LR test of model vs. saturated: chi2(0)   =      0.00, Prob > chi2 =      .

. * intercepts:
. lincom _b[latentch21:1bn.group] - _b[latentch21:2.group]

 ( 1)  [latentch21]1bn.group - [latentch21]2.group = 0

------------------------------------------------------------------------------
             |      Coef.   Std. Err.      z    P>|z|     [95% Conf. Interval]
-------------+----------------------------------------------------------------
         (1) |  -1.613977   4.155301    -0.39   0.698    -9.758217    6.530263
------------------------------------------------------------------------------

. * prop-growth paths:
. lincom _b[latentch21:1bn.group#c.ctotmeth1]- _b[latentch21:2.group#c.ctotmeth1]

 ( 1)  [latentch21]1bn.group#c.ctotmeth1 - [latentch21]2.group#c.ctotmeth1 = 0

------------------------------------------------------------------------------
             |      Coef.   Std. Err.      z    P>|z|     [95% Conf. Interval]
-------------+----------------------------------------------------------------
         (1) |   .3662937   .1983711     1.85   0.065    -.0225066    .7550939
------------------------------------------------------------------------------
```

WITHOUT PROPORTIONAL GROWTH FOR ILLUSTRATION:

```
                        |                 OIM
                        |      Coef.   Std. Err.      z    P>|z|     [95% Conf. Interval]
------------------------+----------------------------------------------------------------
Structural              |
  totmeth2              |
              totmeth1  |
                   [*]  |          1  (constrained)
            latentch21  |
                   [*]  |          1  (constrained)
                 _cons  |
                   [*]  |          0  (constrained)
------------------------+----------------------------------------------------------------
         mean(totmeth1)|
             Guangzhou  |    22.8708    1.56031    14.66   0.000     19.81265    25.92895
               Beijing  |   18.74593   2.129581     8.80   0.000     14.57203    22.91983
       mean(latentch21)|
             Guangzhou  |   4.288648   2.135355     2.01   0.045      .103429    8.473867
               Beijing  |    8.52647    4.09408     2.08   0.037     .5022211    16.55072
------------------------+----------------------------------------------------------------
        var(e.totmeth2)|
                   [*]  |          0  (constrained)
          var(totmeth1)|
             Guangzhou  |   477.1751   48.20197                      391.4655    581.6506
               Beijing  |     390.02   59.47749                      289.2543    525.8888
        var(latentch21)|
             Guangzhou  |   682.1197   78.84195                      543.8455    855.5505
               Beijing  |   1256.091   206.6782                      909.8372    1734.117
------------------------+----------------------------------------------------------------
cov(totmeth1,latentch21)|
             Guangzhou  |  -250.3927   47.38154    -5.28   0.000    -343.2588   -157.5266
               Beijing  |   -347.521   87.87576    -3.95   0.000    -519.7543   -175.2877
-----------------------------------------------------------------------------------------
Note: [*] identifies parameter estimates constrained to be equal across groups.
LR test of model vs. saturated: chi2(0)   =      0.00, Prob > chi2 =      .

.
```

```
. *nlcom _b[/mean(latentch21)#1bn.group] - _b[/mean(latentch21)#2.group]
. lincom _b[/mean(latentch21)#1bn.group] - _b[/mean(latentch21)#2.group]

 ( 1)  [/]mean(latentch21)#1bn.group - [/]mean(latentch21)#2.group = 0

------------------------------------------------------------------------------
             |      Coef.   Std. Err.      z    P>|z|     [95% Conf. Interval]
-------------+----------------------------------------------------------------
         (1) |  -4.237821   4.617492    -0.92   0.359    -13.28794    4.812296
------------------------------------------------------------------------------
```

Work performance

```
. sem (latentch21@1 cjobperf1@1 _cons@0 ->cjobperf2) (cjobperf1 _cons -> latentch21),
group(group) ginvariant(non
> e) nocapslatent latent(latentch21) var(e.cjobperf2@0 e.latentch21 ) method(mlmv) showginvariant
(2 all-missing observations excluded)
note: Missing values found in observed exogenous variables. Using the noxconditional behavior.
Specify the
      forcexconditional option to override this behavior.
Endogenous variables

Observed:  cjobperf2
Latent:    latentch21

Exogenous variables

Observed:  cjobperf1

Structural equation model                       Number of obs     =        280
Grouping variable  = group                      Number of groups  =          2
Estimation method  = mlmv
Log likelihood     = -872.68286

 ( 1)  [cjobperf2]1bn.group#c.latentch21 = 1
 ( 2)  [cjobperf2]1bn.group#c.cjobperf1 = 1
 ( 3)  [/]var(e.cjobperf2)#1bn.group = 0
 ( 4)  [cjobperf2]1bn.group = 0
 ( 5)  [cjobperf2]2.group#c.latentch21 = 1
 ( 6)  [cjobperf2]2.group#c.cjobperf1 = 1
 ( 7)  [/]var(e.cjobperf2)#2.group = 0
 ( 8)  [cjobperf2]2.group = 0
----------------------------------------------------------------------------------
                 |                 OIM
                 |      Coef.   Std. Err.      z    P>|z|     [95% Conf. Interval]
-----------------+----------------------------------------------------------------
Structural       |
  cjobperf2      |
      latentch21 |
             [*] |          1  (constrained)
       cjobperf1 |
             [*] |          1  (constrained)
           _cons |
             [*] |          0  (constrained)
  ---------------+----------------------------------------------------------------
  latentch21     |
       cjobperf1 |
      Guangzhou  |  -.5391502   .0912282    -5.91   0.000    -.7179542   -.3603463
        Beijing  |  -1.241835   .1647798    -7.54   0.000    -1.564797   -.9188723
           _cons |
      Guangzhou  |  -.0327876   .1178219    -0.28   0.781    -.2637142    .1981391
        Beijing  |   .6914081   .2440482     2.83   0.005     .2130825    1.169734
-----------------+----------------------------------------------------------------
  mean(cjobperf1)|
      Guangzhou  |  -.1025018   .1012937    -1.01   0.312    -.3010337    .0960301
```

```
        Beijing  |   .6282801   .1531447     4.10   0.000     .3281221    .9284382
-----------------+----------------------------------------------------------------
 var(e.cjobperf2)|
             [*] |          0  (constrained)
var(e.latentch21)|
      Guangzhou  |   1.952079   .2331145                      1.544714    2.466872
        Beijing  |   2.985658    .504855                      2.143421    4.158843
   var(cjobperf1)|
      Guangzhou  |   1.983593   .2019436                      1.624779    2.421647
        Beijing  |   1.970682   .3040412                      1.456435    2.666501
----------------------------------------------------------------------------------
Note: [*] identifies parameter estimates constrained to be equal across groups.
LR test of model vs. saturated: chi2(0)   =      0.00, Prob > chi2 =      .

. lincom _b[latentch21:1bn.group] - _b[latentch21:2.group]
 ( 1)  [latentch21]1bn.group - [latentch21]2.group = 0

------------------------------------------------------------------------------
             |      Coef.   Std. Err.      z    P>|z|     [95% Conf. Interval]
-------------+----------------------------------------------------------------
         (1) |  -.7241957   .2710009    -2.67   0.008    -1.255348   -.1930436
------------------------------------------------------------------------------
* prop-growth paths:
. lincom _b[latentch21:1bn.group#c.cjobperf1]- _b[latentch21:2.group#c.cjobperf1]

 ( 1)  [latentch21]1bn.group#c.cjobperf1 - [latentch21]2.group#c.cjobperf1 = 0

------------------------------------------------------------------------------
             |      Coef.   Std. Err.      z    P>|z|     [95% Conf. Interval]
-------------+----------------------------------------------------------------
         (1) |   .7026845    .188348     3.73   0.000     .3335292     1.07184
------------------------------------------------------------------------------
```

WITHOUT PROPORTIONAL GROWTH FOR ILLUSTRATION:

```
. sem (latentch21 -> jobperf2@1) (jobperf1@1 _cons@0 ->jobperf2),         group(group)
ginvariant(none) nocapslat
> ent latent(latentch21) means(latentch21 ) var(e.jobperf2@0 latentch21 ) method(mlmv)
showginvariant
(2 all-missing observations excluded)

Endogenous variables

Observed:  jobperf2

Exogenous variables

Observed:  jobperf1
Latent:    latentch21
Structural equation model                        Number of obs     =        280
Grouping variable  = group                       Number of groups  =          2
Estimation method  = mlmv
Log likelihood     = -872.68286

 ( 1)  [jobperf2]1bn.group#c.jobperf1 = 1
 ( 2)  [jobperf2]1bn.group#c.latentch21 = 1
 ( 3)  [/]var(e.jobperf2)#1bn.group = 0
 ( 4)  [jobperf2]1bn.group = 0
 ( 5)  [jobperf2]2.group#c.jobperf1 = 1
 ( 6)  [jobperf2]2.group#c.latentch21 = 1
 ( 7)  [/]var(e.jobperf2)#2.group = 0
 ( 8)  [jobperf2]2.group = 0
----------------------------------------------------------------------------------------
                        |                 OIM
                        |      Coef.   Std. Err.      z    P>|z|     [95% Conf. Interval]
------------------------+---------------------------------------------------------------
```

```
Structural              |
  jobperf2              |
             jobperf1 |
                  [*] |          1  (constrained)
           latentch21 |
                  [*] |          1  (constrained)
                _cons |
                  [*] |          0  (constrained)
----------------------+----------------------------------------------------------------
       mean(jobperf1)|
            Guangzhou  |   6.897498   .1012937    68.09   0.000     6.698966     7.09603
              Beijing  |    7.62828   .1531447    49.81   0.000     7.328122    7.928438
     mean(latentch21)|
            Guangzhou  |   .0224763   .1303576     0.17   0.863      -.23302    .2779726
              Beijing  |   -.088812    .281877    -0.32   0.753    -.6412808    .4636568
----------------------+----------------------------------------------------------------
       var(e.jobperf2)|
                  [*] |          0  (constrained)
        var(jobperf1)|
            Guangzhou  |   1.983593   .2019436                      1.624779    2.421647
              Beijing  |   1.970682   .3040412                      1.456435    2.666501
      var(latentch21)|
            Guangzhou  |   2.528675   .3107017                      1.987489    3.217224
              Beijing  |   6.024752   1.059153                       4.26872    8.503165
----------------------+----------------------------------------------------------------
cov(jobperf1,latentch21)|
            Guangzhou  |  -1.069455   .2112161    -5.06   0.000    -1.483431   -.6554785
              Beijing  |  -2.447261   .4976786    -4.92   0.000    -3.422693   -1.471829
---------------------------------------------------------------------------------------LR test
of model vs. saturated: chi2(0)   =      0.00, Prob > chi2 =      .
. lincom _b[/mean(latentch21)#1bn.group] - _b[/mean(latentch21)#2.group]

 ( 1)  [/]mean(latentch21)#1bn.group - [/]mean(latentch21)#2.group = 0

----------------------------------------------------------------------------
             |      Coef.   Std. Err.      z    P>|z|     [95% Conf. Interval]
-------------+--------------------------------------------------------------
         (1) |   .1112883   .3105604     0.36   0.720    -.4973989    .7199756
----------------------------------------------------------------------------
```

Sitting hours

```
. sem  (latentch21 totsith1@1 _cons@0 ->totsith2) (totsith1 _cons ->latentch21),  gr
> oup(group) ginvariant(none) nocapslatent latent(latentch21)  var(e.totsith2@0 e.la
> tentch21 ) method(mlmv) showginvariant
(3 all-missing observations excluded)
note: Missing values found in observed exogenous variables. Using the
      noxconditional behavior. Specify the forcexconditional option to override
      this behavior.
Endogenous variables

Observed:  totsith2
Latent:    latentch21

Exogenous variables

Observed:  totsith1

Structural equation model                       Number of obs     =        279
Grouping variable  = group                      Number of groups  =          2
Estimation method  = mlmv
Log likelihood     = -1102.5334

 ( 1)  [totsith2]1bn.group#c.latentch21 = 1
 ( 2)  [totsith2]1bn.group#c.totsith1 = 1
```

```
 ( 3)  [/]var(e.totsith2)#1bn.group = 0
 ( 4)  [totsith2]1bn.group = 0
 ( 5)  [totsith2]2.group#c.latentch21 = 1
 ( 6)  [totsith2]2.group#c.totsith1 = 1
 ( 7)  [/]var(e.totsith2)#2.group = 0
 ( 8)  [totsith2]2.group = 0
------------------------------------------------------------------------------------
                 |                 OIM
                 |      Coef.   Std. Err.      z    P>|z|     [95% Conf. Interval]
-----------------+------------------------------------------------------------------
Structural       |
  totsith2       |
      latentch21 |
             [*] |          1  (constrained)
        totsith1 |
             [*] |          1  (constrained)
           _cons |
             [*] |          0  (constrained)
  ---------------+------------------------------------------------------------------
  latentch21     |
        totsith1 |
      Guangzhou  |  -.5851763   .0826396    -7.08   0.000    -.7471469   -.4232056
        Beijing  |  -1.101411   .1097504   -10.04   0.000    -1.316518   -.8863041
           _cons |
      Guangzhou  |     5.6775   .8156443     6.96   0.000     4.078867    7.276134
        Beijing  |   10.34172   1.037181     9.97   0.000      8.30888    12.37456
-----------------+------------------------------------------------------------------
   mean(totsith1)|
      Guangzhou  |   9.507772   .1934689    49.14   0.000      9.12858    9.886964
        Beijing  |    9.20747   .2403564    38.31   0.000      8.73638     9.67856
-----------------+------------------------------------------------------------------
  var(e.totsith2)|
             [*] |          0  (constrained)
var(e.latentch21)|
      Guangzhou  |   5.499059   .6693242                      4.33195    6.980609
        Beijing  |   3.996969   .6806905                      2.86266    5.580741
    var(totsith1)|
      Guangzhou  |   7.224033   .7353872                     5.917383    8.819211
        Beijing  |   4.851632   .7485811                     3.585518    6.564833
------------------------------------------------------------------------------------
Note: [*] identifies parameter estimates constrained to be equal across groups.
LR test of model vs. saturated: chi2(0)   =      0.00, Prob > chi2 =      .

. * intercepts:
. lincom _b[latentch21:1bn.group] - _b[latentch21:2.group]

 ( 1)  [latentch21]1bn.group - [latentch21]2.group = 0

------------------------------------------------------------------------------
             |      Coef.   Std. Err.      z    P>|z|     [95% Conf. Interval]
-------------+----------------------------------------------------------------
         (1) |  -4.664218   1.319478    -3.53   0.000    -7.250346    -2.07809
------------------------------------------------------------------------------

. * prop-growth paths:
. lincom _b[latentch21:1bn.group#c.totsith1]- _b[latentch21:2.group#c.totsith1]

 ( 1)  [latentch21]1bn.group#c.totsith1 - [latentch21]2.group#c.totsith1 = 0

------------------------------------------------------------------------------
             |      Coef.   Std. Err.      z    P>|z|     [95% Conf. Interval]
-------------+----------------------------------------------------------------
         (1) |   .5162347   .1373844     3.76   0.000     .2469663    .7855031
------------------------------------------------------------------------------
```
WITHOUT PROPORTIONAL GROWTH FOR ILLUSTRATION:

```
. sem  (latentch21 totsith1@1 _cons@0 ->totsith2),       group(group) ginvariant(no
> ne) nocapslatent latent(latentch21) means(latentch21 ) var(e.totsith2@0 latentch21
>  ) method(mlmv) showginvariant
(3 all-missing observations excluded)

Endogenous variables

Observed:  totsith2

Exogenous variables

Observed:  totsith1
Latent:    latentch21
Structural equation model                       Number of obs     =        279
Grouping variable  = group                      Number of groups  =          2
Estimation method  = mlmv
Log likelihood     = -1102.5334

 ( 1)  [totsith2]1bn.group#c.totsith1 = 1
 ( 2)  [totsith2]1bn.group#c.latentch21 = 1
 ( 3)  [/]var(e.totsith2)#1bn.group = 0
 ( 4)  [totsith2]1bn.group = 0
 ( 5)  [totsith2]2.group#c.totsith1 = 1
 ( 6)  [totsith2]2.group#c.latentch21 = 1
 ( 7)  [/]var(e.totsith2)#2.group = 0
 ( 8)  [totsith2]2.group = 0
---------------------------------------------------------------------------------
                |                 OIM
                |      Coef.   Std. Err.      z    P>|z|     [95% Conf. Interval]
----------------+----------------------------------------------------------------
Structural      |
  totsith2      |
       totsith1 |
            [*] |          1  (constrained)
     latentch21 |
            [*] |          1  (constrained)
          _cons |
            [*] |          0  (constrained)
----------------+----------------------------------------------------------------
  mean(totsith1)|
     Guangzhou  |   9.507772   .1934689    49.14   0.000      9.12858    9.886964
       Beijing  |    9.20747   .2403564    38.31   0.000      8.73638     9.67856
mean(latentch21)|
     Guangzhou  |   .1137779   .2314559     0.49   0.623    -.3398674    .5674232
       Beijing  |   .2005102   .3573284     0.56   0.575    -.4998406     .900861
----------------+----------------------------------------------------------------
 var(e.totsith2)|
            [*] |          0  (constrained)
   var(totsith1)|
     Guangzhou  |   7.224033   .7353872                      5.917383    8.819211
       Beijing  |   4.851632   .7485811                      3.585518    6.564833
 var(latentch21)|
     Guangzhou  |   7.972794    .999788                       6.23548    10.19415
       Beijing  |   9.882513   1.629717                      7.153136    13.65332
----------------+----------------------------------------------------------------
   cov(totsith1,|
     latentch21)|
     Guangzhou  |  -4.227333   .7359236    -5.74   0.000    -5.669716   -2.784949
       Beijing  |   -5.34364   .9811248    -5.45   0.000    -7.266609   -3.420671
---------------------------------------------------------------------------------
Note: [*] identifies parameter estimates constrained to be equal across groups.
LR test of model vs. saturated: chi2(0)   =      0.00, Prob > chi2 =      .

. *nlcom _b[/mean(latentch21)#1bn.group] - _b[/mean(latentch21)#2.group]
```

```
. lincom _b[/mean(latentch21)#1bn.group] - _b[/mean(latentch21)#2.group]

 ( 1)  [/]mean(latentch21)#1bn.group - [/]mean(latentch21)#2.group = 0

------------------------------------------------------------------------------
             |      Coef.   Std. Err.      z    P>|z|     [95% Conf. Interval]
-------------+----------------------------------------------------------------
         (1) |  -.0867323    .425741    -0.20   0.839    -.9211694    .7477048
------------------------------------
```

5. Mixture/latent class SKEWT illustration from Mplus

```
---3-----------T LCS model--------Sitting-------------------
NS.C > NS.I, NS.Diff
SYNTAX for SKEWT Mplus model:
Usevariables are  !
     y2 y1 ;
    IDVARIABLE = id;
    CLASSES = c1t2 (2) ;
            KNOWNCLASS = c1t2 (group = 1 group = 2);
    Define:
    y1 = ctotsith1; !centered on PRE C=BJ mean
    y2 = ctotsith2; !centered on PRE C=BJ mean
    ANALYSIS: TYPE = MIXTURE;
              COVERAGE = 0;
              DISTRIBUTION = SKEWT;! SKEW SKEWT normal tdistribution
              STARTS = 32 8; ! typically not necessary
              PROCESSORS = 8;
    Model:
    %OVERALL%
     LCS by y2@1;
      y2@0;
      [y2@0];
      [LCS] ;
       LCS on y1;
     y2 on y1@1;
        !y1 on ;! covariates possible
        !LCS on tot1 ;! proportional growth path; also covariates possible

    %c1t2#1%
       LCS by y2@1;
      y2@0;
      [y2@0];
      [LCS] (CLCSInt);
       LCS on y1;
     y2 on y1@1;
        !y1 on ;! covariates possible
        !LCS on tot1 ;! proportional growth path; also covariates possible

    %c1t2#2%
         LCS by y2@1;
      y2@0;
      [y2@0];
      [LCS] (TLCSInt);
       LCS on y1;
     y2 on y1@1;
        !y1 on ;! covariates possible
        !LCS on tot1 ;! proportional growth path; also covariates possible

    Model constraint:
    New (TE);
    TE=TLCSInt-CLCSInt; !T-C difference tested for significance

THE MODEL ESTIMATION TERMINATED NORMALLY



MODEL FIT INFORMATION

Number of Free Parameters                       10

Loglikelihood

          H0 Value                        -564.172
```

```
          H0 Scaling Correction Factor      1.1269
            for MLR
Information Criteria
          Akaike (AIC)                    1148.343
          Bayesian (BIC)                  1181.426
          Sample-Size Adjusted BIC        1149.744
            (n* = (n + 2) / 24)
FINAL CLASS COUNTS AND PROPORTIONS FOR THE LATENT CLASSES
BASED ON THE ESTIMATED MODEL
    Latent
   Classes
!EC: these are actually observed classes/known
       1        135.00000          0.66832
       2         67.00000          0.33168
MODEL RESULTS
                                                       Two-Tailed
                    Estimate       S.E.  Est./S.E.    P-Value

Latent Class 1 (1) = INTERVENTION
 LCS      BY
    Y2                 1.000      0.000    999.000    999.000
 LCS        ON
    Y1                -0.517      0.169     -3.057      0.002
 Y2         ON
    Y1                 1.000      0.000    999.000    999.000
 Intercepts
    Y2                 0.000      0.000    999.000    999.000
    LCS                0.014      0.586      0.024      0.981
 Residual Variances
    Y2                 0.000      0.000    999.000    999.000
    LCS                2.186      0.575      3.799      0.000

Latent Class 2 (2) = CONTROLS
 LCS      BY
    Y2                 1.000      0.000    999.000    999.000
 LCS        ON
    Y1                -1.097      0.064    -17.162      0.000
 Y2         ON
    Y1                 1.000      0.000    999.000    999.000
 Intercepts
    Y2                 0.000      0.000    999.000    999.000
    LCS               -0.304      0.680     -0.448      0.654
 Residual Variances
    Y2                 0.000      0.000    999.000    999.000
    LCS                2.186      0.575      3.799      0.000

Categorical Latent Variables
 Means
    C1T2#1             0.701      0.149      4.688      0.000
!EC: MPLUS TESTS FOR SKEW THE DV/EFFECT VARIABLES ONLY, AND THE LCAS MODEL SEEMS TO SHOW THE LCS
SCORE FOR PA IS NOT SKEWED
Skew and Df Parameters
Latent Class 1 (1)
    LCS                0.353      0.662      0.534      0.594
    DF                 2.868      0.840      3.412      0.001
Latent Class 2 (2)
    LCS                0.863      0.807      1.069      0.285
    DF                 5.013      2.731      1.836      0.066
New/Additional Parameters
    TE                -0.318      0.839     -0.380      0.704
```

<u>IF ONE TESTS ONLY tdistribution BY CHANGING ONLY:</u>

```
DISTRIBUTION = tdistribution;
MODEL RESULTS
                                                       Two-Tailed
```

```
                      Estimate       S.E.  Est./S.E.    P-Value

Latent Class 1 (1) = INTERVENTION
 LCS      BY
    Y2                 1.000      0.000    999.000    999.000
 LCS        ON
    Y1                -0.530      0.168     -3.165      0.002
 Y2         ON
    Y1                 1.000      0.000    999.000    999.000
 Intercepts
    Y2                 0.000      0.000    999.000    999.000
    LCS                0.337      0.206      1.634      0.102
 Residual Variances
    Y2                 0.000      0.000    999.000    999.000
    LCS                2.451      0.494      4.966      0.000

Latent Class 2 (2) = CONTROLS
 LCS      BY
    Y2                 1.000      0.000    999.000    999.000
 LCS        ON
    Y1                -1.085      0.068    -16.056      0.000
 Y2         ON
    Y1                 1.000      0.000    999.000    999.000
 Intercepts
    Y2                 0.000      0.000    999.000    999.000
    LCS                0.441      0.241      1.832      0.067
 Residual Variances
    Y2                 0.000      0.000    999.000    999.000
    LCS                2.451      0.494      4.966      0.000
Categorical Latent Variables
 Means
    C1T2#1             0.701      0.149      4.688      0.000
Skew and Df Parameters
Latent Class 1 (1)
    DF                 3.108      0.812      3.825      0.000
Latent Class 2 (2)
    DF                 5.332      3.099      1.721      0.085
New/Additional Parameters
    TE                 0.104      0.305      0.341      0.733
```

Home (../) Academy (https://clincalc.com/academy/) Blog (../blog) About (/About.aspx)

(/Default.aspx)

(/Default.aspx)

6. Power analysis exhibit from https://clincalc.com/stats/samplesize.aspx

Appendix Move-It: A cluster-randomised digital worksite exercise intervention

≡ Menu

Sample Size Calculator

Determines the minimum number of subjects for adequate study power

ClinCalc.com (/) » Statistics (/Statistics) » Sample Size Calculator

Study Group Design

vs. Two independent study groups

vs. One study group vs. population

Two study groups will each receive different treatments.

Primary Endpoint

Back to Top

Dichotomous
(yes/no)

Continuous
(means)

The primary endpoint is **binomial** - only two possible outcomes.
Eg, mortality (dead/not dead), pregnant (pregnant/not)

Statistical Parameters

Anticipated Means

Group 1 ? 5 ± 15

Group 2 ? 12

Mean ▼

Enrollment ratio ? 1

Type I/II Error Rate

Alpha ? 0.05

Power ? 80%

Reset **Calculate**

Back to Top
Top

RESULTS

Continuous Endpoint, Two Independent Sample Study

Sample Size	
Group 1	72
Group 2	72
**Total**	**144**

Study Parameters	
Mean, group 1	5
Mean, group 2	12
Alpha	0.05
Beta	0.2
Power	0.8

View Power Calculations

About This Calculator

This calculator uses a number of different equations to determine the minimum number of subjects that need to be enrolled in a study in order to have sufficient statistical power to detect a treatment effect.[1]

Before a study is conducted, investigators need to determine how many subjects should be included. By enrolling too few subjects, a study may not have enough statistical power to detect a difference (type II error). Enrolling too many patients can be unnecessarily costly or time-consuming.

Back to Top
Top

Generally speaking, statistical power is determined by the following variables:

- **Baseline Incidence:** If an outcome occurs infrequently, many more patients are needed in order to detect a difference.
- **Population Variance:** The higher the variance (standard deviation), the more patients are needed to demonstrate a difference.
- **Treatment Effect Size:** If the difference between two treatments is small, more patients will be required to detect a difference.
- **Alpha:** The probability of a type-I error -- finding a difference when a difference does not exist. Most medical literature uses an alpha cut-off of 5% (0.05) -- indicating a 5% chance that a significant difference is actually due to chance and is not a true difference.
- **Beta:** The probability of a type-II error -- not detecting a difference when one actually exists. Beta is directly related to study power (Power = 1 - β). Most medical literature uses a beta cut-off of 20% (0.2) -- indicating a 20% chance that a significant difference is missed.

Post-Hoc Power Analysis

To calculate the post-hoc statistical power of an existing trial, please visit the **post-hoc power analysis calculator (Power.aspx)**.

References and Additional Reading

1. Rosner B. *Fundamentals of Biostatistics*. 7th ed. Boston, MA: Brooks/Cole; 2011.

Search

Enter search term ...

Related Calculators

- Post-hoc Power Calculator (Power.aspx)

Follow Us!

(http://www.facebook.com/clincalc) (http://twitter.com/clincalc) (http://feeds.feedburner.com/ClinCalc)

Back to Top

Top

MEDCALC®
easy-to-use statistical software

7. Test of differences in attrition from https://www.medcalc.org/calc/comparison_of_proportions.php Appendix Move-It: A cluster-randomised digital worksite exercise intervention 1

Free statistical calculators

Comparison of proportions calculator

Sample 1

Proportion (%): 27

Sample size: 196

Sample 2

Proportion (%): 15.1

Sample size: 86

Test

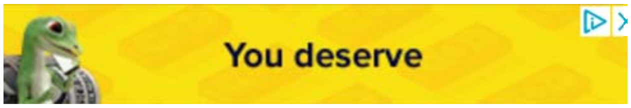

Results

Difference	11.9 %
95% CI	1.1934% to 20.8701%
Chi-squared	4.710
DF	1
Significance level	P = 0.0300

Computational notes

MedCalc uses the "N-1" Chi-squared test as recommended by Campbell (2007) and Richardson (2011).

The confidence interval is calculated according to the recommended method given by Altman et al. (2000).

Literature

- Altman DG, Machin D, Bryant TN, Gardner MJ (Eds) (2000) Statistics w 49)

This site uses cookies to store information on your computer. More info... OK

- Campbell I (2007) Chi-squared and Fisher-Irwin tests of two-by-two tables with small sample recommendations. Statistics in Medicine 26:3661-3675. PubMed
- Richardson JTE (2011) The analysis of 2 x 2 contingency tables - Yet again. Statistics in Medicine 30:890. PubMed

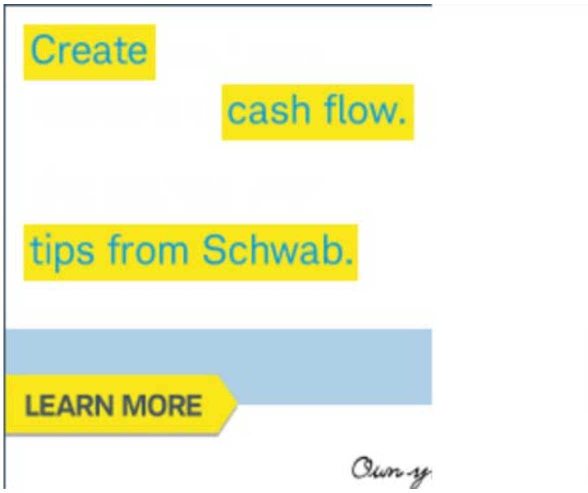

© 2019 MedCalc Software bv

This site uses cookies to store information on your computer. More info... OK
